# Acceptability of Computerized Cognitive Behavioral Therapy for Adults: Umbrella Review

**DOI:** 10.2196/23091

**Published:** 2021-07-06

**Authors:** Charlene J Treanor, Anne Kouvonen, Tea Lallukka, Michael Donnelly

**Affiliations:** 1 Centre for Public Health Queen's University Belfast Belfast United Kingdom; 2 Faculty of Social Sciences University of Helsinki Helsinki Finland; 3 Department of Public Health University of Helsinki Helsinki Finland

**Keywords:** computerized/internet cognitive behavioral therapy, cCBT, iCBT, acceptability, mental health, umbrella review

## Abstract

**Background:**

Mental ill-health presents a major public health problem. A potential part solution that is receiving increasing attention is computer-delivered psychological therapy, particularly during the COVID-19 pandemic as health care systems moved to remote service delivery. However, computerized cognitive behavioral therapy (cCBT) requires active engagement by service users, and low adherence may minimize treatment effectiveness. Therefore, it is important to investigate the acceptability of cCBT to understand implementation issues and maximize potential benefits.

**Objective:**

This study aimed to produce a critical appraisal of published reviews about the acceptability of cCBT for adults.

**Methods:**

An umbrella review informed by the Joanna Briggs Institute (JBI) methodology identified systematic reviews about the acceptability of cCBT for common adult mental disorders. Acceptability was operationalized in terms of uptake of, dropping out from, or completion of cCBT treatment; factors that facilitated or impeded adherence; and reports about user, carer, and health care professional experience and satisfaction with cCBT. Databases were searched using search terms informed by relevant published research. Review selection and quality appraisal were guided by the JBI methodology and the AMSTAR tool and undertaken independently by 2 reviewers.

**Results:**

The systematic searches of databases identified 234 titles, and 9 reviews (covering 151 unique studies) met the criteria. Most studies were comprised of service users with depression, anxiety, or specifically, panic disorder or phobia. Operationalization of acceptability varied across reviews, thereby making it difficult to synthesize results. There was a similar number of guided and unguided cCBT programs; 34% of guided and 36% of unguided users dropped out; and guidance included email, telephone, face-to-face, and discussion forum support. Guided cCBT was completed in full by 8%-74% of the participants, while 94% completed one module and 67%-84% completed some modules. Unguided cCBT was completed in full by 16%-66% of participants, while 95% completed one module and 54%-93% completed some modules. Guided cCBT appeared to be associated with adherence (sustained via telephone). A preference for face-to-face CBT compared to cCBT (particularly for users who reported feeling isolated), internet or computerized delivery problems, negative perceptions about cCBT, low motivation, too busy or not having enough time, and personal circumstances were stated as reasons for dropping out. Yet, some users favored the anonymous nature of cCBT, and the capacity to undertake cCBT in one’s own time was deemed beneficial but also led to avoidance of cCBT. There was inconclusive evidence for an association between sociodemographic variables, mental health status, and cCBT adherence or dropping out. Users tended to be satisfied with cCBT, reported improvements in mental health, and recommended cCBT. Overall, the results indicated that service users’ preferences were important considerations regarding the use of cCBT.

**Conclusions:**

The review indicated that “one size did not fit all” regarding the acceptability of cCBT and that individual tailoring of cCBT is required in order to increase population reach, uptake, and adherence and therefore, deliver treatment benefits and improve mental health.

## Introduction

Globally, mental health problems and mental disorders are a major public health concern [1]. Collectively, the results of multiple studies and systematic reviews over the last few decades appear to point to the overall effectiveness of cognitive behavioral therapy (CBT) [2,3]. CBT aims to help individuals overcome, change, and challenge aberrant thought processes and behaviors [4]. The advent of the internet and surge in the use of computer technology led to the delivery of CBT via computer- or web-based platforms, with demonstrable effectiveness to treat and manage mental health conditions and symptoms [5-7]. Computerized delivery of CBT (cCBT) is a generic term that encompasses web-based or internet-delivered CBT (iCBT). It can be delivered in the community, at home, or in a health care setting and can be self-directed or self-guided by the user or is guided via telephone or email by a health care professional or practitioner [8]. There is evidence to suggest that cCBT may be as effective as face-to-face delivered CBT [9,10], and, for example, the UK National Institute for Health and Clinical Excellence (NICE) recommends the use of cCBT for specific conditions such as mild depression [11]. Compared to face-to-face CBT, cCBT may remove or minimize barriers associated with uptake and be more accessible (eg, for rural dwellers and individuals who have mobility issues) and affordable; thereby, it may reduce waiting times and be more cost-effective [12,13]. In addition, the absence of in-person contact with cCBT delivery may reduce stigma associated with mental health service use for some individuals even though perceptions towards help-seeking for mental health in many countries have taken a positive step over recent years [14].

The onset of a global pandemic of the SARS-CoV-2 virus occurred during the conduct of this umbrella review. Government-directed measures designed to control the transmission of the virus such as social distancing, quarantine, and self-isolation were implemented widely. These measures necessitated a move to remote-delivered therapies [15].

Mental health interventions such as CBT require active engagement from participants in order to affect therapeutic change. Adherence and the degree to which an individual engages with, and completes, a web-based intervention is a potential limitation. Poor adherence limits exposure to a web or computerized program, and an insufficient “dose” may impact treatment effectiveness and reduce the likelihood of an improved outcome for people with mental health problems, particularly if they drop out of a program. It is important to enhance understanding about acceptability and adherence in order to gain insights about the implementation of cCBT and to increase its effectiveness [16]. Improving understanding about acceptability is even more important now given that mental disorders and mental health problems appear to be increasing due to the direct and indirect effects of COVID-19 and the possibility that remotely delivered psychological therapies may become the norm in the context of strategies designed to minimize virus transmission [17]. The results of our initial scoping activity indicated that there has been an exponential increase in the number of systematic reviews and there are international collaborations that specialize in systematic reviews and maintain databases of systematic reviews. Therefore, we decided to synthesize the evidence from existing systematic reviews regarding the acceptability of cCBT by undertaking a review of reviews or an “umbrella” review [18]. We used cCBT to encompass also iCBT, as some programs are delivered without the internet (eg, CD-ROMs), and our focus was on mental health generally to encompass experiences with both psychological symptoms and clinically diagnosed disorders. Acceptability [19] was defined broadly as encompassing factors that facilitate or impede uptake, adherence, and completion or that contribute to attrition (including reported reasons for dropping out) and satisfaction. This review also captured service users’ and therapists’ or clinicians’ views of cCBT [19].

## Methods

The search strategy for the umbrella review [18] was informed by the use of the following key concepts from published reviews: “systematic reviews,” “cCBT,” “iCBT,” “barriers,” “facilitators,” and “common mental disorders and symptoms” [20-22]. The Boolean operators “AND” and “OR” were used to combine search terms between and within concepts, respectively. The PubMed, EMBASE, MEDLINE, PsycINFO, and CINAHL databases were searched from inception until December 18, 2019 (see Textbox 1). Titles and abstracts were imported into an Excel spreadsheet, and duplicates were removed by CT. MD and CT independently assessed each title and abstract against the eligibility criteria and independently applied the Joanne Briggs Institute critical appraisal checklist for systematic reviews [18], supplemented with one item from the AMSTAR (A Measurement Tool to Assess Systematic Reviews) checklist [23]. Any disagreements regarding inclusion of articles were resolved through discussion. Results of the quality appraisal checklist are reported in Multimedia Appendix 1. Review papers were included in the umbrella review if they met the criteria that are detailed in the following sections.

**MEDLINE search strategy (adapted for other databases).**

#1 ((internet or web or online) adj3 (cognitive or behavio*)).ti,ab,kf. OR (iCBT or i-CBT or ePsych* or e-Psych or cCBT or c-CBT).ti,ab,kf

#2 computer communication networks/ or internet/ or blogging/ or social media/ OR cell phones/ or smartphone/ or text messaging/ or videoconferencing/ or webcasts as topic/ or wireless technology/ OR Telemedicine/ OR (eLearning or blended learning).ti,kf. OR (videoconferenc* or video conferenc*).ti,kf OR (synchronous or asynchronous or (electronic adj2 deliver*)).ti,kf. OR android.ti,ab,kf. OR (app or apps or blog*).ti,ab,kf. OR (cell phone or cellphone or chat room or computer* or cyber* or digital or technology based or DVD).ti,ab,kf. OR CD-ROM.ti,ab,kf. OR (eHealth or electronic health or email*).ti,ab,kf. OR (ePortal or eTherap* or forum* or gaming or information technolog* or instant messag* or messaging or internet* or ipad or iphone or ipod or podcast or smart phone or smartphone or social network* site* or social networking or mHealth or mobile or multimedia or online* or personal digital assistant or PDA or SMS or social medi* or software or telecomm* or telehealth* or telemed* or telemonitor* or telepsych* or teletherap* or text messag* or texting or virtual* or web* or WWW).ti,ab,kf,hw.

#3 (behavio* or cognitive).ti. OR ((cognitive or behavio*) adj2 (activat* or component? or defusion or modif* or restructur* or technique* or intervention or treatment* or therap* or train*)).mp. OR (psychotherap* or psychological therap* or cognitive behavio*).mp. OR ((acceptance* or commitment*) adj3 therap*).mp. OR (rational emotive or RET or problem sol* or PST or problem focus* or solution focus* or trauma focus* or psychoeducat* or psychodrama or mindfulness* or third wave or self control).mp. OR (self* adj3 (control or efficacy)).mp. OR (stress manage* or exposure or reality therap*).mp. OR (anxiety adj3 (management or therap* or train*)).mp. OR (relaxation or guided imagery or present cent* or person cent* or person* construct* or therapeutic process* or schema? or schemata).mp. OR (thought* adj3 suppress*).mp. OR rumination.mp.

#4 #2 AND #3

#5 #1 OR #4

#6 “Systematic Review”/ OR systematic review.ti,ab,kf.

#7 #5 AND #6

#8 program evaluation.mp. OR program evaluation/ OR process evaluation.mp. OR “process assessment (health care)”/ OR (process evaluation* OR qualitative component* OR qualitative aspect* OR qualitative approach* OR systematic evaluation* OR participant observation OR simulation OR implementation audit).mp. OR (audit OR feedback).tw. OR qualitative research/ OR qualitative.mp. OR qualitative research.mp.

#9 #7 AND #8

#10 depression/ OR depression.mp. OR anxiety/ OR anxiety.mp. OR Obsessive-Compulsive Disorder/ OR obsessive compulsive disorder.mp. OR Mental Disorders/ OR common mental disorders.mp. OR Occupational Stress/ OR Stress Disorders, Post-Traumatic/ OR stress.mp. OR Stress, Psychological/ OR mental health.ti OR mental illness.ti. OR psychiatric.ti OR mood disorder.tw OR neurotic disorder.sh

#11 #9 AND #10

### Population and Focus of the Review

The target population was people with common mental disorders or experiencing an increase in psychological symptoms (eg, depression, anxiety, and distress) [1]. Systematic reviews of studies of mixed populations and interventions were included if it was possible to disaggregate data in relation to the use of cCBT to target psychological symptoms or mental disorders. Reviews that addressed the topic of acceptability including reviews of quantitative and qualitative studies were included, and reviews that assessed effectiveness only were excluded unless they investigated one or more aspects of acceptability. Systematic reviews only were included as, usually, they include a focused research question with defined parameters and follow an a priori agreed methodology. Other review types such as scoping reviews were excluded because, typically, they tend to address a broad review question and, often, are limited due to time and scoping restraints [24].

### Intervention

Reports of interventions had to indicate that that they were grounded in CBT in terms of, for example, aiming to improve or maintain mental health outcomes by changing aberrant thought patterns and behaviors. CBT interventions delivered via the internet or via computer only were included [4]. Interventions were included if they were guided (by email, telephone, or online therapist support) or self-directed/unguided (no therapist support) and accessed in a clinical, community, or home-based setting. The duration of an intervention or the intensity with which it was delivered was not used as a criterion for excluding a review.

### Outcomes

The scoping activity and the review team discussions identified the following key outcomes or indicators of acceptability: percentage of uptake or refusal following invitation to cCBT; adherence (number of completed intervention sessions or components); proportion of “drop-outs” (including reasons for, time of, and factors associated with dropping out); satisfaction with intervention components or mode of delivery; and user, carer, or health care professional reports about their views of cCBT.

There were no language nor year restrictions. Three non-English language papers were identified, but from examining available English abstracts, they did not meet the review criteria. Double counting of primary studies across reviews is an inherent bias of an umbrella review, and where present, efforts have been made to indicate study overlap. Some reviews included physical health conditions and non-cCBT interventions, and these findings were excluded from the umbrella review.

### Data Extraction

We followed best guidance on the conduct of umbrella reviews [18] and studied examples of the methodological approach that were reported in other umbrella reviews [25]. Data were extracted by CT and checked by MD. The following data were extracted into tabular format: first author, publication year, study design, research question, searches, type of study and setting, participant details, type of intervention, quality appraisal method and scores, and research synthesis method. Thus, data were synthesized narratively at review level and under the following headings: uptake or refusal, adherence or cCBT completion, proportion and timing of “drop-outs,” satisfaction or acceptability of cCBT, and users’ views of cCBT (including therapists’ views).

## Results

**Figure 1:**
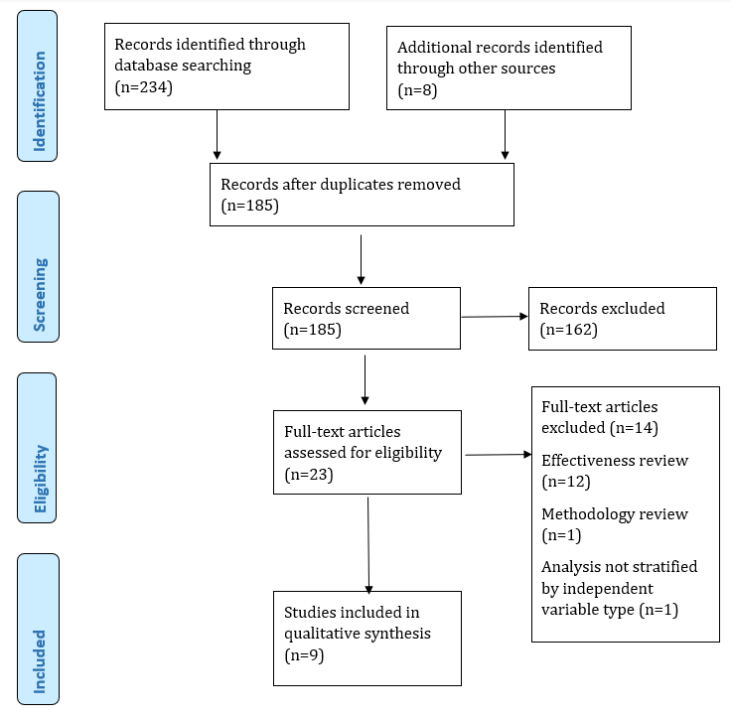
Preferred Reporting Items for Systematic Reviews and Meta-Analyses (PRISMA) flowchart.

The database searches identified 234 titles: 23 full-text papers remained following duplicate removal and abstract screening, and a further 8 titles were identified through citation lists of the 23 reviews. Of the 31 papers, 9 met the eligibility criteria for inclusion in the umbrella review (see Figure 1 for the Preferred Reporting Items for Systematic Reviews and Meta-​Analyses [PRISMA] flowchart). The 9 reviews covered 151 unique studies, with 27 of the 151 studies reported in more than one review. Individual studies varied in terms of study design including experimental design (n=111), qualitative studies (n=15), mixed methods (n=8), feasibility studies (n=7), cohort or survey design (n=4), and a systematic review (n=1). This systematic review was not identified during the electronic searches because it did not meet one or more of the concepts that informed the search strategy and it focused on the effectiveness of online mental health programs rather than directly addressing the umbrella review question. Regarding reviews that included intervention studies, most control groups were waitlist (n=21), were attention controls (n=13), received no treatment (n=12), or received treatment as usual (n=10).

Most review studies focused on adult populations (n=119), while a smaller number examined adolescent populations (n=8) and one study included both adults and adolescents [26]. One review [27] did not report the target population, and so, 7 of the 16 primary studies in this review were examined (9/16 studies were not accessible because of paywall restrictions). Studies covered a range of mental disorders and symptoms including depression (n=80), anxiety (n=24), panic disorder or phobia (n=26), stress or distress (n=9), insomnia (n=6), posttraumatic stress disorder (n=4), eating disorders (n=4), alcohol misuse disorders (n=3), grief (n=2), body image issues (n=1), bipolar disorder (n=1), and unspecified or nondefined “public health mental disorders” (n=1). Some studies covered more than one mental health condition. Reviews focused on individuals with clinically diagnosed disorders [26,28-30], experiencing psychological symptoms [31,32], or both [27,33,34]. We refer throughout the review, where possible, to whether findings relate to mental disorders or symptoms. Individuals with physical conditions or other populations (eg, cancer caregivers, survivors of natural disasters) were the focus of 9 studies, and details about the populations in 5 studies were not provided (see Multimedia Appendix 2).

### Intervention Characteristics

We examined 178 cCBT interventions across the reviews, with 23 studies examining more than one cCBT condition. Two reviews were not focused only on cCBT and included a small number of non-cCBT interventions (n=1) or did not specify the type of therapeutic intervention (n=2). Detailed descriptions about interventions including their content, format, and delivery were reported infrequently. It was possible to discern, where this information was reported, that there were 53 guided and 50 unguided interventions; guidance included email support (n=11), telephone support (n=9), face-to-face contact (n=9), and a discussion forum (n=3). Technical support was provided in 25 studies (see Multimedia Appendix 2).

### Uptake or Refusal

Data on the proportion of people in each study who availed of the use of a cCBT intervention were provided in 5 reviews. Definitions of uptake varied widely and referred to qualitatively different types of individuals; for example, 1 review included the proportion of eligible individuals who were randomized and spontaneous users who accessed cCBT websites [26]. A very wide range of uptake proportions (1%-97%) was reported across reviews [26-29,34]. Only 1 review reported the proportion of people who refused (9%-24%) [26]. In 2 reviews, the proportions who enrolled to receive cCBT and take part in its evaluation but then did not take up cCBT varied widely from 1% to 63% [26,29] (see Tables 1 and 2). Rates of uptake, refusal, or not starting were not separated by type of cCBT (eg, self-guided or guided) in reviews, and often this information was not reported by reviews for individual cCBT programs. All 5 reviews included studies of people with depression or anxiety.

**Table 1:** Adherence definitions and rates.

Study	Adherence definition	Rates of uptake	Proportion completing treatment^a^	Drop-outs
Kaltenthaler et al (2008) [34]^b^	Patient recruitment; number of patients who accessed website and agreed to take part in study; drop-outs; number of patients who dropped out of the study	25% agreed to take part	Type of program (% participants did not complete components, number of studies): Beating the Blues (26%-45%, 5 studies); MoodGym (17%-75%, 2 studies) website mass recruitment; ODIN (34%, 2 studies); COPE (29%-32%, 2 studies); Recovery Road (32%, 1 study); Five Areas Approach (30%, 1 study); BALANCE (11%, 1 study); two unnamed interventions (0%, 37%)	Mean 31.75% (SD 16.52%); range 0%-75%
Waller and Gilbody (2009) [27]^c^	Invited: those who received information about the study; recruited: those specifically asked to take part in the study; finished study: % of those who started and completed the study; finished modules: % of those who completed all modules	Median 38%; range 4%-84%	Started and subsequently completed: median 83%, range 26%-100%; overall completion: median 56%, range 12%-100%	Individuals in cCBT were twice as likely to drop-out than control groups (OR 2.03, 95% CI 0.81-5.09)
Melville et al (2010) [30]^d^	Drop-out: “…leaving treatment before its completion…at one of a number of points throughout treatment”; pretreatment drop-out: before beginning of treatment; treatment dropout: prior to completion of treatment sessions; follow-up drop-out: prior to completing follow-up assessments; rates of drop-out; number of eligible participants (denominator) and the number of individuals who terminated at any point from registering the treatment and completing follow-up questionnaires (numerator)	Not reported	Not reported	Pretreatment drop-out: 4%-52% (median 10%; weighted median^e^ 21%); treatment dropout: 0%-78% (median 10%; weighted median 21%); follow-up drop-out: 0%-18% (median 4%; weighted median 8%)
Vallury et al (2015) [26]^f^	Rates of uptake and rates of adherence	Uptake rate: 44%-86%; refusal rate: 9%-24%; did not start: 7%	Completed treatment: 33%-100%; completed follow-up at 3 months: 63%	Not reported
Beatty and Binnion (2016) [29]^g^	Quantitative studies: adherers were those who completed program or completed the posttreatment assessment; nonadherers were those who did not complete program or did not complete the posttreatment assessment; numbers of modules/sessions/assessments completed, duration of logins, time using program, number of logins, number of homework assignments completed, accessing the program; qualitative studies: those who do not complete treatment, barriers to adherence	Uptake rate: 41.3%; did not start: 1%-63%	Completed all sessions: 16.9%-83.0%, 35.8%-66.0% for unguided or unsupported interventions, 58.0%-74.4% for guided or supported interventions; completed only one session or module: 27%-90%, 94% for guided, 95% for unguided; completed some but not all sessions or modules: 10%-99%, 67%-84% for guided interventions, 54.4%-93.0% for unguided interventions; completed all assessments: 26.0%-90.8%	Early drop-out (during first sessions): 10.0%-56.9%, 37.1% for the treatment group, 32.1% for the control group; drop-outs at end of study: 4.25%-38.00%, 56.3%-75.0% for the treatment group, 29%-48% for the control group, 35.9% for guided, 33.7% for unguided, 56% of those who immediately accessed, 80% of those who delayed access
Rost et al (2017) [28]^h^	Uptake, drop-out, or completion rates as a means of assessing user acceptance	39%-97%	Mean 67.17% (SD 20.29%), range 26.7%-100%; 8.1%, 56% guided intervention; 16.28%, 36% unguided intervention	Mean 31.5% (SD 19.49%), range 0%-63%
Twomey and O’Reilly 2017 [32]^i^	Proportion of participants withdrawing before final data collection, proportion of individuals who completed intervention	Not reported	10%-100%	Withdrew before posttreatment data collection: 0%-64%

^a^This was defined differently across reviews; in some cases, this included proportions of those who started and subsequently completed treatment or follow-up assessments.

^b^Of the 16 studies, 10 were overlapping.

^c^Of the 36 studies, 15 were overlapping.

^d^Of the 19 studies, 6 were overlapping.

^e^Weighted for study sample size.

^f^Of the 11 studies, 1 was overlapping; 4 studies included adolescent populations.

^g^Of the 36 studies, 6 were overlapping; 6 studies included populations with physical health or other conditions and thus were excluded.

^h^Of the 29 studies, 6 were overlapping; 2 studies included adolescents.

^i^Of the 11 studies, 5 were overlapping.

**Table 2:** Findings from quantitative studies (as categorized by review) referring to factors associated with adherence or dropping out.

Factor	Intervention (population)	Number of studies or participants	Results or findings (number of studies)	Heterogeneity^a^
**Demographic variables (not specified)**				
	Internet-based cognitive behavioral therapy (CBT; subthreshold depression)	8 studies	No difference between completers and noncompleters [31]^b^	No association
**Gender**				
	Internet-based treatment (psychological dysfunction or distress related to psychiatric conditions)	2 studies	No association (1); men more likely to drop-out (1) [30]^c^	Mixed results
	Self-directed psychological intervention (psychological outcomes for mental and physical disorders)	15 studies	Women more likely to adhere (7), men more likely to adhere (1), no association with gender (7) [29]^b^	Not reported
**Age**				
	Internet-based treatment (psychological dysfunction or distress related to psychiatric conditions)	2 studies	No association (1)^d^, younger age associated with drop-out (1) [30]^c^	Mixed results
	Self-directed psychological intervention (psychological outcomes for mental and physical disorders)	14 studies	No association (6)^d^, older age associated with adherence (4), younger age associated with adherence (3), mixed findings (1) [29]^b^	Not reported
**Education level**				
	Internet-based treatment (psychological dysfunction or distress related to psychiatric conditions)	2 studies	No association (2 [1]^d^) [30]^c^	No association
	Self-directed psychological intervention (psychological outcomes for mental and physical disorders)	13 studies	No association (8)^d^, higher education associated with higher adherence (4), lower education associated with higher adherence (1) [29]^b^	Not reported
**Ethnicity**				
	Self-directed psychological intervention (psychological outcomes for mental and physical disorders)	1 study	No association [29]^b^	Not enough evidence
**Being partnered**				
	Internet-based treatment (psychological dysfunction or distress related to psychiatric conditions)	1 study	Being partnered associated with drop-out (1) [30]^c^	No association
	Self-directed psychological intervention (psychological outcomes for mental and physical disorders)	8 studies	No association with having a partner (6), being partnered associated with adherence (2) [29]^b^	No association
**Employment status**				
	Computerized CBT (cCBT; mild or moderate depression)	1 study	Getting a job provided as reason for dropping out (2) [34]^c^	No association
	Self-directed psychological intervention (psychological outcomes for mental and physical disorders)	7 studies	No association (7) [29]^b^	Not reported
**Geographical location**				
	Self-directed psychological intervention (psychological outcomes for mental and physical disorders)	1 study	Oceania or Europe residency associated with higher completion of modules than residency in North America, South America, and Africa (1) [29]^b^	Not enough evidence
**Residency (urban/rural)**				
	cCBT (prevention of anxiety and depression)	2 studies	Adherence and/or attrition among rural compared to urban participants, rurality had no effect (1) or negative effect (1) on retention to cCBT [26]^b,c^	Not enough evidence
	Self-directed psychological intervention (psychological outcomes for mental and physical disorders)	2 studies	Within Ireland and Australia, no association (2) [29]^b^	Not reported
**Personal circumstances**				
	cCBT (mild or moderate depression)	2 studies	Family reasons (3) or change in circumstances (15%) or moving house (10%) provided as reasons for dropping out [34]^c^	Limited evidence
	cCBT, (common mental health disorders)	9 studies (126 participants)	Personal circumstances were stated as a reason for declining^d^ [27]^c^	Not reported
**Time commitments**				
	cCBT (mild or moderate depression)	2 studies	Being too busy (8) provided as reason for dropping out [34]^c^	Limited evidence
	cCBT (depression)	6 studies	Lack of time reported (6) as reason for dropping out [28]^c^	Not reported
**Physical health**				
	cCBT (mild or moderate depression)	1 study	Ill-health (15%) provided as reason for dropping out of study [34]^c^	Not enough evidence
**Mental health status variables (unspecified)**				
	Internet-based CBT (subthreshold depression)	8 studies	No difference between completers and noncompleters [31]^b^	Not enough evidence
**Symptom severity**				
	cCBT (mild or moderate depression)	1 study	Improvement in symptoms was reported as a reason for dropping out (2)^e^ [34]^c^	Mixed evidence
	cCBT (common mental health disorders)	2 studies (2 participants)	Perceived increased risk (potential increase in symptoms) reported as reasons for drop-out ^d^ [27]^c^	Mixed evidence
	Internet-based treatment (psychological dysfunction or distress related to psychiatric conditions)	3 studies	Lower symptom severity associated with dropping out of study (3 [1]^d^) [30]^c^	Not reported
	Self-directed psychological intervention (psychological outcomes for mental and physical disorders)	20 studies	At baseline: no association (10), lower symptom severity associated with increased adherence (6) and increased module completion (1); higher symptom severity associated with higher adherence (3) [29]^b^	Not reported
**Duration of problem**				
	cCBT (mild or moderate depression)	1 study	Improvement in condition (10%) provided as reason for dropping out of study [34]^c^	Mixed evidence
	Internet-based treatment (psychological dysfunction or distress related to psychiatric conditions)	2 studies	No association (2) [30]^c^	Not reported
	Self-directed psychological intervention (psychological outcomes for mental and physical disorders)	3 studies	Longer duration associated with higher adherence (2), no association (1) [29]^b^	Not reported
**Psychiatric diagnosis**				
	Internet-based treatment (psychological dysfunction or distress related to psychiatric conditions)	2 studies	Comorbid depression and anxiety no association (2) [30]^c^	Not enough evidence
	Self-directed psychological intervention (psychological outcomes for mental and physical disorders)	4 studies	Diagnosis of anxiety or depression associated with higher adherence (3), and alcohol dependency associated with higher adherence among waitlist control group (1) [29]^b^	Not enough evidence
**Medication or alcohol use**				
	Self-directed psychological intervention (psychological outcomes for mental and physical disorders)	2 studies	No association (2; studies not targeting alcohol dependency) [29]^b^	Not enough evidence
**Treatment credibility or expectation**				
	cCBT (mild or moderate depression)	4 studies	Perception that intervention is not useful (11), unhelpful (10), didn’t like treatment (n not reported), inappropriate for needs (1) provided as reason for dropping out of study^e^ [34]^c^	Mixed evidence
	cCBT (common mental health disorders)	11 studies (101 participants)	Therapy was reported as a reason for dropping out, but it is not clear what this meant or which group it referred to [27]^c^	Not reported
	Internet-based treatment (psychological dysfunction or distress related to psychiatric conditions)	2 studies	No association (2) [30]^c^	Not reported
	cCBT (prevention of anxiety and depression)	1 study	Treatment preference fulfilment was associated with adherence to the study for rural residing participants [26]^b^	Not reported
	Self-directed psychological intervention (psychological outcomes for mental and physical disorders)	9 studies	Positively associated with higher adherence (7), no association (2) [29]^b^	Not reported
	cCBT (depression)	6 studies	Treatment being perceived as inconvenient (4) was reported as a reason to drop out [28]^c^	Not reported
**Motivation and readiness to change**				
	cCBT (mild or moderate depression)	2 studies	Low motivation (8), inability to commit (n not reported), and no desire to continue (n not reported) provided as reasons for dropping out [34]^c^	Limited evidence
	Self-directed psychological intervention (psychological outcomes for mental and physical disorders)	4 studies	Treatment readiness associated with higher adherence (2), and intention to complete treatment associated with higher adherence (1); intention to complete treatment no association (1) [29]^b^	Not reported
**Self-efficacy or self-confidence**				
	Self-directed psychological intervention (psychological outcomes for mental and physical disorders)	3 studies	No association (2), taking responsibility for one’s own choices was associated with higher adherence to a bulimia self-guided program (1) [29]^b^	Not enough evidence
**Computer-related issues**				
	cCBT (mild or moderate depression)	2 studies	Internet-related issues (5), changed mind about PC delivery (1) provided as reasons for dropping out [34]^c^	Mixed evidence
	cCBT (common mental health disorders)	5 studies (14 participants)	Information technology issues were not commonly reported as a reason for dropping out [27]^c^	Not reported
	Self-directed psychological intervention (psychological outcomes for mental and physical disorders)	4 studies	Higher adherence was associated with website usability (1) and a positive attitude to a computerized self-guided format (1), no association between adherence and computer literacy level (2) [29]^b^	Not reported
	cCBT (depression)	4 studies	Computer or technical issues (4) were reported as reasons for dropping out of the study [28]^c^	Not reported
**Guidance or therapist support**				
	cCBT (mild or moderate depression)	1 study	Preference for face-to-face help (8) provided as reason for dropping out [34]^c^	Guided intervention associated with higher adherence
	Self-directed psychological intervention (psychological outcomes for mental and physical disorders)	8 studies	Guided interventions were associated with higher adherence than unguided interventions (4), phone support was associated with higher adherence than email support (1), no difference in adherence between guided and unguided interventions (3) [29]^b^	Not reported
**Referral source**				
	Self-directed psychological intervention (psychological outcomes for mental and physical disorders)	3 studies	General practitioner referral (2) or the media (1) associated with higher adherence [29]^b^	Not enough evidence
**Program content**				
	cCBT (mild or moderate depression)	2 studies	Other help sought provided as reason for dropping out (2), treatment not demanding (n not reported) [34]^c^	Not enough evidence
	Self-directed psychological intervention (psychological outcomes for mental and physical disorders)	2 studies	Gratitude intervention group was twice as likely to complete treatment than a monitoring and restructuring intervention group (1); tailored feedback to increase self-efficacy, personalization of intervention team (eg, photo with “we”) increased adherence for participants who accessed all intervention components (1) [29]^b^	Not reported
**Setting**				
	cCBT (mild or moderate depression)	2 studies	Hard to attend (13) or journey too long (3) provided as reasons for dropping out [34]^c^	Not enough evidence
**Duration of intervention**				
	cCBT (mild or moderate depression)	16 studies	Duration influenced drop-out (direction of effect not reported in review); duration of interventions ranged from 1-33 sessions; the authors note that it is diﬃcult to make comparisons between cCBT programs regarding drop-out rates because of diﬀerences in study design, populations, and methods for deﬁning drop-outs and level of detail provided in a study [34]^c^	Duration associated with adherence
**Group membership**				
	cCBT (mild or moderate depression)	1 study	Intervention group had higher drop-outs compared to participants in the information website group [34]^c^	Control groups more likely to adhere than intervention groups
	cCBT (common mental health disorders)	9 studies	Participants in cCBT were twice as likely to drop out than participants in the control group (OR 2.03, 95% CI 0.81-5.09) [27]^c^	Not reported
	Self-directed psychological intervention (psychological outcomes for mental and physical disorders)	4 studies	Control (waitlist) group membership predicted higher adherence than intervention group membership (1), intervention group membership associated with adherence (1), no association with group membership (2) [29]^b^	Not reported

^a^At least 50% of studies needed to provide evidence of or absence of an association; there was not enough evidence if <5 studies available.

^b^Association with adherence.

^c^Association with drop-out.

^d^Overlapping studies.

^e^Number of participants (where reported in reviews).

### Adherence to or Completion of One or More Intervention Sessions or Components

The definitions of adherence varied between and within reviews. For example, some reviews included studies that defined adherence in terms of “completers” or individuals who completed an outcome assessment at the end of receipt of cCBT while other studies defined “treatment completers” as individuals who completed all “modules” or a specified “dose” of cCBT even if they did not undertake a research outcome assessment [29]. The proportion of participants who completed an entire cCBT program ranged from 10% to 100% [26-29,32]. Only 1 review [29] provided more detailed data about adherence — the proportion of individuals who discontinued after 1 session or module fluctuated between 27% and 90%, and completion of more than 1 session or module ranged from 10% to 99%.

Heterogeneity in terms of the proportion of individuals who completed cCBT was present for guided and unguided cCBT (guided cCBT: completed full program, 8%-74%; completed 1 module only, 94%; completed some modules, 67%-84%; and unguided cCBT: completed full program, 16%-66%; completed 1 module only, 95%; completed some modules, 54%-93%) [28,29]. The proportions of cCBT users who remained in studies after completion of cCBT and until follow-up assessments were infrequently reported [26,29] and probably provide more evidence for the acceptability of research participation rather than cCBT acceptability.

### Dropping Out or Discontinuing Use of cCBT

Two reviews reported (without providing details about timing) that, on average, around one-third of users “dropped out” [28,34]. According to 2 reviews, service users dropped out pretreatment, before cCBT started (range 4%-52%) [30], during cCBT treatment (range 0%-78%) [30], “early” within the first few sessions (10%-57%) [29], and during posttreatment (0%-38%) [29,30]. A wide range of cCBT participants (0%-64%) did not remain in cCBT studies for posttreatment data collection [32].

The proportion of service users who dropped out of guided (36%) and unguided (34%) cCBT programs was similar [29]. One review reported that cCBT participants in a treatment group were twice as likely to drop out than active attention control participants [27]. However, this finding was neither significant nor supported by a second review [29], which compared “early” drop-outs within the first few sessions (treatment group, 37% vs control group, 32%) to individuals who dropped out at the end of cCBT (treatment group, range 56%-75% vs control group, range 29%-48%).

### Sociodemographic Factors and Adherence

Across 2 reviews, 7 of 16 studies (and 1 overlapping study) reported the absence of an association between age and adherence [29,30]. The remaining 9 studies in the 2 reviews found mixed evidence (1/16) or that older age (5/16) or younger age (3/16) was associated with cCBT adherence or dropping out. Regarding gender across the same 2 reviews [29,30], there was a positive association between being female and adherence (8/16 studies) or no association (8/16 studies), and 1 study concluded that men were more likely to adhere than female participants. Overall, the evidence from the reviews indicated that there was no association between education [29,30], having a significant other [29,30], or employment status [29,34] with adherence or dropping out. One review [31] stated (without providing supporting data) that there were no sociodemographic differences between people who completed or did not complete cCBT. The limited available evidence did not indicate that ethnicity [29], geography [29], or urban or rural residency [26,29] played a role in adherence (see Table 2).

### Mental Health Status–Related Factors and Adherence

The association between mental health symptom severity and adherence was unclear [27,29,30,34]. Across 4 reviews, 10 of 26 studies reported no association between symptom severity and adherence; 9 studies observed that adherence was sustained when fewer and less severe symptoms were experienced while 7 studies reported an association between experiencing more symptoms and adherence. The relationship between duration of symptoms and adherence across 3 reviews [29,30,34] was inconsistent (no association: 3/6 studies; shorter duration association: 1/6 studies; and longer duration association: 2/6 studies). In 1 review [29], 2 studies found no association between adherence and medication or alcohol use, respectively; an examination of attrition bias (as part of risk of bias assessment) indicated that there were no mental health differences between people who completed and did not complete cCBT [31]. The relationship between physical health status and adherence is unknown (see Table 2).

### cCBT Features and Adherence

cCBT features and adherence were examined in 4 reviews. Guided compared to unguided cCBT was associated with higher adherence (4/7 studies), or there was no association (3/7) [29]. Adherence was better sustained via telephone compared to email support, though only 1 review addressed the type of support [29]. A preference for face-to-face therapy compared to cCBT was stated as a reason for dropping out for 8/60 participants (only 39 participants stated reasons) in 1 study [34]. Internet or computerized delivery problems were cited as reasons for dropping out in 2 of 3 reviews [28,34]. There was no association between computer literacy and adherence (2 studies), though cCBT website competency (1 study) and a positive attitude towards computerized delivery of CBT (1 study) were associated with higher adherence [29]. There was wide variation in the duration of cCBT programs (range 1-33 sessions). Only 1 review tested the relationship between cCBT duration and adherence, reporting that duration of cCBT was a factor in dropping out [34]. Higher adherence (or fewer drop-outs) was observed in control groups compared with cCBT programs [27,29,34]. A meta-analysis of 9 studies [27] reported that controls may be twice as likely as cCBT participants to drop out of studies (OR 2.03, 95% CI 0.81-5.09). The reviews did not investigate other factors such as the role of referral source, specific program content, delivery setting, access, and adherence (see Table 2). Most were narrative reviews and did not include or included only minimal statistical results.

### Behavioral Factors and Adherence

Behavioral factors and adherence were examined in 6 reviews. Negative perceptions about cCBT (eg, as unhelpful or inappropriate) were associated with dropping out [28,34], while positive expectations were associated with good adherence [26,29]. The direction of the relationship between cCBT expectancies and adherence was uncertain or absent in 2 reviews [27,29,30]. Low motivation was a reason for dropping out in 1 review (2 studies) [34], and a second review [29] found that intending or being ready to participate in cCBT was associated with higher adherence (3/4 studies). Several studies across 3 reviews reported that being too busy or not having enough time (8 studies) [28,34] and “personal circumstances” (10 studies) [27,34] were reasons for dropping out. The role of self-efficacy and adherence was investigated by a limited number of studies [29] (see Table 2).

### Service Users’ Views

Generally, users appeared to be highly satisfied [26-28,34] and would recommend cCBT [27,34]; mixed reports of satisfaction were infrequently reported by individual review studies [28]. Rural-dwelling participants were more likely than urban participants to report that cCBT improved their depression or substance misuse and were more likely to report that they were satisfied with cCBT support and liked the autonomy, confidentiality, and privacy that it afforded [26]. The anonymous nature of remotely delivered cCBT was preferred compared to face-to-face therapies [28], though some users reported feelings of increased isolation [28] and uncertainty about the privacy of cCBT [29]. The capacity to undertake cCBT in your own time was perceived to be beneficial [27-29] but also led to avoidance of cCBT [27,28]. Similarly, being too busy or unable to find the time to undertake cCBT was reported in several studies [29]. Users experienced difficulties finding a quiet, private space to access a computer to undertake cCBT [30]. The computerized format (and associated technical aspects) was reported to be easy to use, particularly when training was provided [27,34]. User perceptions of their information technology (IT) skills or openness to using computers impeded adherence [27-30], and the additional time that older participants required due to lower technical competency appeared to be off-putting [27].

Predominantly based on qualitative data (with the exception of quantitative data in 1 review [34]), guided cCBT programs appeared to lead to greater adherence [27,28,34]. Studies that compared guided and unguided cCBT were mixed or unclear regarding satisfaction and perceived helpfulness [28]. There was a preference for face-to-face CBT [27,30], and generally users who had experience with both modes perceived cCBT as more beneficial [27], though some studies reported criticisms by users in terms of cCBT lacking human contact and sufficient guidance [29]. The role that therapists played in guided cCBT or face-to-face CBT was perceived positively because they had particular skills (eg, promoting understanding of condition or therapy) or characteristics (eg, more tolerant) or were considered more helpful for particular patient groups (eg, complex mental health cases) [27]. A small number of reviews reported service users’ views about some aspects of cCBT programs. The use of a booklet, multimedia design, and program structure [34] and program design [27] were noted positively by users. There was a positive association between adherence and when cCBT programs were viewed as helpful versus when programs were perceived as impersonal or irrelevant [28,29]. One review [27] reported that particular user groups had different levels of motivation and adherence levels (eg, self-referrals [higher] and mental health referrals [lower]), and delivery of cCBT in a primary care setting was viewed positively (see Multimedia Appendix 3).

A qualitative review containing a meta-synthesis identified 2 “core constructs” to explain factors that impeded or facilitated participation in cCBT: “sensitivity of self and identity” and “the dialectical nature of user experience.” First, the preferences, needs, and challenges that an individual with mental ill-health faces (eg, reduced motivation and concentration) need to be considered before deciding that cCBT is an appropriate option or to inform how to tailor the delivery of cCBT. Second, the delivery of cCBT needs to take into account the contradictory presence of perceived benefits and drawbacks (eg, the option to self-select modules may be empowering and burdensome, and cCBT may be perceived as enhancing confidentiality and privacy or as an obstacle to meeting the need for face-to-face support from therapists and peers) [33]. Lack of motivation related to depression was a barrier to cCBT adherence, and feeling that cCBT was not helping was related to poor adherence [27]. Some users appeared to drop out of cCBT programs because they were perceived to be too general, limited, or intensive or they had negative experiences with particular cCBT components [29]. Therapists’ views about cCBT were captured by 1 study in 1 review only [27]. Their views revolved around the following factors: the availability of resources (the need for training, computer suite, and costs); institutional support to provide resources; “culturally tailored” cCBT; cCBT as an adjunct to, rather than a replacement for, face-to-face therapy; concerns about security; and data protection. Finally, therapists rated face-to-face therapy as safer and more effective [27] (see Multimedia Appendix 4).

## Discussion

### Principal Findings

This umbrella review investigated the acceptability of cCBT, in terms of the proportion of people who availed of, adhered to, or dropped out of cCBT as well as with reference to qualitative reports of the reasons for dropping out and users’ and therapists’ views. Nine reviews provided relevant data, though the aforementioned indicators of acceptability were defined and operationalized heterogeneously across reviews. The extent of this variation made it difficult to “sum up” the results from the 9 reviews and to compare acceptability across populations, conditions, programs, and settings. Several reviews reported that cCBT retention proportions (eg, face-to-face CBT drop-out range, 5%-38% and cCBT mean drop-out, 33%) were comparable and as variable as psychological therapy delivered face-to-face [27,28,30,34], thereby suggesting that acceptability of cCBT may be on par with face-to-face therapies and unrelated to features of computerized delivery.

Often, reasons were not given for dropping out of studies, so it is difficult to draw firm conclusions regarding why adherence to cCBT was difficult for some individuals. It is important to understand the reasons for dropping out and whether they vary at different stages. For example, did individuals withdraw because cCBT was not what they expected, treatment was too intensive, symptoms improved (whether or not improvement was attributed to cCBT), or for other reasons separately or in combination. Included reviews did not present data regarding why individuals chose not to take up cCBT at all or that illuminated a deeper understanding about reasons for dropping out. Developing strategies to increase uptake, adherence, and retention with respect to cCBT requires a clear understanding about the factors associated with adherence or reasons for dropping out. Most reviews gathered quantitative data, and it is likely that a mixed methods research approach would help to advance our understanding about the reasons for problems and challenges. Indeed, the quantitative results were inconsistent regarding sociodemographic variables, health status, cCBT characteristics, and behavioral factors and their association with cCBT adherence.

The qualitative synthesis presented by Knowles et al [33] stressed the importance of taking into account individuals’ preferences and captured the contradictory nuances related to user acceptability of cCBT. The results from the quantitative studies indicated that guided cCBT was associated with better adherence, whereas qualitative findings indicated that there were favorable and unfavorable aspects to guided and unguided cCBT formats. Very few reviews directly compared guided and unguided formats, and like-with-like comparisons were not prominent. There is a need for future research to consider whether there are issues that are specific to particular types of cCBT. Targeted training may help to ease apprehensions about IT, and improving computer literacy may overcome reluctance to participate in cCBT.

There is a need for further research to improve understanding about “dosage” and dropping out. The review points clearly to the conclusion that “one size does not fit all” and that cCBT even in relatively small “doses” may work well for some patients including patients who drop out at various stages, whereas a “full dose” and perhaps even more again is needed by other patients. For example, a recent randomized controlled trial and intention-to-treat analysis found a beneficial effect for the cCBT treatment of insomnia despite a high drop-out rate and increasingly lower adherence to modules over the course of the study. It is important to note that the study was comprised of a student population and most students experienced subclinical insomnia [35,36]. It was not possible to discern from our umbrella review which types of patients benefited from different “doses” of cCBT, and there are similar unanswered questions for “brief” face-to-face CBT and its variants.

Only limited attention was given to the potential for theory to illuminate understanding about the acceptability of cCBT [28,30] such as diffusion of innovations theory [37] and the Technology Acceptance Model [38], particularly concepts around perceptions about the ease of use and the effectiveness of cCBT. The use of theories of implementation science (eg, the Consolidated Framework for Implementation Research [39]) may also facilitate understanding about acceptability and the implementation of cCBT within a health care system, particularly if health care professionals or therapists are resistant to delivering cCBT. The limited evidence in the umbrella review regarding therapist acceptability found fewer positive views about cCBT. Acceptability and implementation may be achieved if cCBT is delivered within a “stepped care model” in which individuals begin with face-to-face therapy and then transfer to a cCBT “step,” though there may be complex cases that require ongoing individual face-to-face therapy [27]. There may be ethical concerns that require consideration (eg, avoiding the transfer of burden from health care professionals to service users [27] and possibly creating inequalities around internet access [40-42] or due to education level).

### Limitations

Though a protocol was developed for this review, it was not registered on a registry such as Prospero. However, this umbrella review was informed by an established rigorous methodology for the “summing” of the increasing number of systematic reviews about a given topic and involves independent screening, appraisal, and data extraction. There was a consensus among review authors that study or program heterogeneity and variability and inadequate reporting of details in primary studies restricted meaningful analytical comparisons between cCBT programs, and we relied upon authors’ reports of study and data type (eg, qualitative or quantitative) and use of validated outcome measures of acceptability or satisfaction. Moreover, there is a lot of variance in terms of the labels and descriptions used to describe internet or computerized interventions such as cCBT, and this lack of consensus may contribute to difficulties in research synthesis [43]. This limitation of umbrella review methodology was compounded further due to the reliance on reviews as a secondary data source rather than searching through primary studies to try to identify missing details. It is important to remain cognizant that self-selection of participants to primary research studies (eg, more women) may influence adherence and reports of cCBT experience. This review did not apply any language restrictions, though only English-language reviews met the review criteria. Primary studies published in other languages could provide information about the acceptability of translated versions of cCBT or perhaps for migrant populations where language barriers may exist. The use of a reporting guideline such as the Template for Intervention Description and Replication (TIDieR) Checklist [44] or even use of relevant parts (eg, Part 5 – Interventions) of the CONSORT (Consolidated Standards of Reporting Trials) extension for reporting studies of social and psychological interventions [45] would be a major advance towards achieving a reliable synthesis and furthering our understanding about the acceptability of cCBT.

### Conclusions

Collectively, the results of the reviews indicate that “one size does not fit all” regarding the acceptability of cCBT and that individual patient and service user group tailoring of cCBT are required to increase the population reach and uptake of cCBT and adherence and so, deliver treatment benefits and improve mental health. In turn, tailoring to create as close a patient-treatment match as possible is likely to involve a blending process involving a human guide or therapist.

## Appendix

Multimedia Appendix 1 Supplementary table 1: quality appraisal.

Multimedia Appendix 2 Supplementary table 2: included review characteristics.

Multimedia Appendix 3 Supplementary table 3: satisfaction and acceptability.

Multimedia Appendix 4 Supplementary table 4: qualitative findings.

## Abbreviations

AMSTAR: A Measurement Tool to Assess Systematic Reviews
CBT: cognitive behavioral therapy
cCBT: computerized cognitive behavioral therapy
CONSORT: Consolidated Standards of Reporting Trials
iCBT: internet-delivered cognitive behavioral therapy
IT: information technology
NICE: National Institute for Health and Clinical Excellence
PRISMA: Preferred Reporting Items for Systematic Reviews and Meta-​Analyses
TIDiER: Template for Intervention Description and Replication
