# Supplementary material for: Acceptability of Computerized Cognitive Behavioral Therapy for Adults: Umbrella Review
Source: JMIR Ment Health. 2021 Jul 6;8(7):e23091. doi: 10.2196/23091 (PMC8292944; doi:10.2196/23091)
Supplement: Multimedia Appendix 1 [file mental_v8i7e23091_app1.docx]

**Supplementary Table 1: Quality Assessment results**

| Criterion  Study ID | Clear and explicitly stated review question | Appropriate inclusion criteria to address research question | Appropriate search strategy | Adequate sources and resources for searching | Appropriate criteria for appraising studies | Critical appraisal conducted by two or more independent reviewers | Methods to minimise errors in data extraction | Appropriate methods used to combine studies | Publication bias assessed | Policy and/or practice supported by reported data | Appropriate direction for new research | AMSTAR item: characteristics of included studies provided |
| --- | --- | --- | --- | --- | --- | --- | --- | --- | --- | --- | --- | --- |
| Beatty  2016 [26] | Yes | Yes | Yes | Yes | Yes | No | No | Yes | No | Yes | Yes | Yes |
| Kaltenhaler 2008 [31] | Yes | Yes | Yes | Yes | No | No | No | No | No | Yes | Yes | Yes |
| Knowles  2014 [30] | Yes | Yes | Yes | Yes | Yes | Yes | Yes | Yes | No | Yes | Yes | Yes |
| Melville  2010 [27] | Yes | Yes | Yes | Yes | No | No | No | Yes | No | Yes | Yes | Yes |
| Rost  2017 [25] | Yes | Yes | Yes | Yes | No | No | Yes | Yes | No | Yes | Yes | Yes |
| Twomey  2017 [29] | Yes | Yes | Yes | Yes | Yes | Yes | No | Yes | Yes | Yes | Yes | Yes |
| Vallury  2015 [23] | Yes | Yes | Yes | Yes | Yes | Yes | No | Yes | No | Yes | Yes | Yes |
| Waller  2009 [24] | Yes | Yes | Yes | Yes | Yes | Yes | Yes | Yes | Yes | Yes | Yes | Yes |
| Zhou  2016 [28] | No | Yes | Yes | Yes | Yes | Yes | Yes | Yes | No | Yes | Yes | Yes |
