# Supplementary material for: Acceptability of Computerized Cognitive Behavioral Therapy for Adults: Umbrella Review
Source: JMIR Ment Health. 2021 Jul 6;8(7):e23091. doi: 10.2196/23091 (PMC8292944; doi:10.2196/23091)
Supplement: Multimedia Appendix 2 [file mental_v8i7e23091_app2.docx]

**Supplementary Table 2: Included review characteristics^a^**

|  | Objective and type of review | Search strategy and primary studies details | Participant details | Type of intervention | Appraisal | Research synthesis |
| --- | --- | --- | --- | --- | --- | --- |
| Beatty 2016 | *Objective*  To summarise the available quantitative and qualitative data on characteristics that predict adherence and participant-reported reasons for adhering to online self-directed psychological interventions  *Type of review*  A systematic review (quantitative and qualitative data | *Search terms*  adherence, internet-based, self-help and treatment  *Number and type of databases searched* PsycINFO, Medline and CINAHL  Other sources of study identification- Citation lists of included studies  Search years range of each database searching - 2000 to 2015  Publication year range of included studies – 2002 – 2015  *Number of studies included*  36 (32 focussed on CBT)  *Study design*  Intervention studies (25), mixed method design (7), feasibility studies (3), cohort studies (1) and qualitative (1)  *Setting/*  *context or geographic location*  Unspecified location of access. Delivery: online (33 studies), computer (1), phone and email (1), online and email (1) | *Type of mental health problems*  Targeting psychological outcomes for a mental or physical health condition.  Depression/anxiety (13 studies), insomnia (5), bulimia nervosa (3), social anxiety disorder (3), alcohol disorders (2), bipolar disorder (1), body satisfaction (1), stress (1), smoking cessation (1), public health mental health disorders (1). Remaining studies were physical health disorders or natural disaster survivors  The number of participants across all studies 102,263 (range 13- 82,159)  Age – Adults 18 years old and over. Mean age 39.7 years old  Gender - women 38-100%  Ethnicity –not reported;  SES – unspecified  Computer literacy – not reported;  Exclusions – unspecified | Any ‘…internet-based self-guided psychosocial therapeutic intervention…’  ‘…self-guided interactive categorised into educational, self-guided therapeutic or human-supported therapeutic web-based interventions…producing cognitive, affective and behavioural changes; are typically based on empirically supported face-to-face treatments; and require active engagement…information-only and is considered therapeutically inactive’  CBT delivered in 32 studies, problem-solving (1) ACT (1) unspecified (2)  Staff support needed: unguided (15 studies), guided (11), both (7), unspecified (2), self-guided (1)  Supplementary support to cCBT intervention: unspecified | *Instrument*  Five criteria for empirically supported psychotherapies outlined by Chambless and Hollon (1988) covering i) appropriate study design with control group, ii) adequate sample size (minimum of 25 participants per group), iii) specified inclusion criteria (including targeted population), iv) use of valid and reliable outcome measures and v) appropriate data analysis  *Rating of quality analysis*  Dichotomous rating of ‘met’ or ‘unmet’ for each criterion. All criteria met (13 studies), appropriate control conditions (23), adequate sample size (33), clear participant inclusion criteria (32), valid and reliable measures (30) and appropriate data analysis (19) | *Type*  Narrative synthesis  *Method*  Participants were categorised on a continuum of ‘low’, ‘high’ or ‘non-adherers (dropouts)’ or dichotomously as ‘adherers’ or ‘dropouts’ according to study definitions. Predictors were summarised as follows: demographic characteristics, presenting problem-related factors, psychological factors and intervention/computer factors. Within each category, predictors were summarised in decreasing order based on the number of studies, which addressed each predictor; quantitative and qualitative data were presented separately. Evidence was summarised using the following criteria: ‘yes’ if ≥50% of studies found evidence for the predictor, ‘no’ if ≥50% of studies found evidence against the predictor and ‘unclear’ if more than 5 studies found mixed evidence for the predictor, and ‘inconclusive’ if less than 5 studies assessed the predictor. |
| Kaltenhaler 2008 | *Objective*  Do patients with (mild to moderate) depression (with or without anxiety) accept computerised CBT (cCBT)?  *Type of review*  A systematic review | *Search terms*  Depression were combined with intervention terms such as cognitive therapy AND computer. Names of cCBT packages were also searched as text words. Unpublished studies (obtained from authors of relevant studies identiﬁed in the searches) were included if relevant outcome data were reported.  *Number and type of databases searched* Fifteen databases (eg Medline, PsycINFO, CINAHL, EMBASE and grey literature).  Reference lists of relevant articles were checked and health services research-related resources (eg HTA organisations, guideline-producing bodies, research and trials registers and specialist mental health sites) were scanned.  Search years range of each database searching - inception to July 2005  *Publication year range of included studies* – 1966 to June 2007  *Number of studies* included: 16  *Study design*  RCTs (8), non-RCTs (1), cohort study (7)  *Setting/*  *context or geographic location*  Internet CBT was accessed from a patient’s or service user’s home in 6/16 studies. The delivery settings in the remaining 10/16 studies included an occupational health service, GP/primary care, psychology/community mental health centre and a hospital | *Type of mental health problems*  Not reported specifically - patients with (mild to moderate) depression (with or without anxiety). The HTA tables report that there were studies of participants with panic/phobias (as well as depression/anxiety) though the tables in the report do not read across in a precisely matched data way to the tables in the paper. Details about the various recruitment methods indicated that some participants were not formally diagnosed and came from the general population (3702/5162; 72% - 2794 of 3702 participated in CBT via the MoodGYM programme on its website) whilst other participants were referred by a health professional or had received a formal diagnosis of depression (1460/5162; 28%)  Only very limited details reported. The linked-HTA report presents patient characteristics in Tables 27 and 28. However, there is a mismatch between some of the studies listed in the tables and the 16 studies that are reported in the journal paper. The number of participants across the 16 studies summed to 5162 and ranged from 20 to 274 with one outlier reporting 2794 in terms of website visitors.  Age – not reported; gender – not reported; ethnicity –not reported; SES – not reported; mixed urban/rural – not reported; computer literacy – not reported; exclusions – not reported (but see results below | “CBT (as defined in the studies) delivered alone or as part of a package of care either via a computer interface (personal computer or the internet) or over the telephone with a computer response.”  6/16 studies were described explicitly as internet programmes (eg Beating the Blues) that were accessed from a patient’s or service user’s home though one iCBT programme was facilitated via primary care/clinical psychology service. Most programmes that were accessed in health settings appeared to be the same programmes that potentially could be accessed at home via the internet: Beating the Blues (5 studies), MoodGym (2), Overcoming Depression on the Internet (2), No name (2), Recovery Road (1) and the Five areas approach (1). An interactive computer-aided telephone system called COPE was use in two studies and a CD-ROM called BALANCE was used in the remaining study.  Staff support needed: not specified though it is likely that there was staff support available for pts who accessed a cCBT programme in a health setting (eg primary care).  Supplementary support to cCBT intervention : not specified; cCBT did not appear to be situated within ‘stepped-care’ mental health provision. | *Instrument*  Not reported in the paper – the HTA report specifies the CASP checklist for RCTs and the Downs and Black checklist for non-RCTs.  *Rating of quality analysis*  Not reported. Study quality is described in Tables 21 and 22 in the HTA report based on the questions in the respective appraisal checklists. However, I could not find any reference to whether or not the results of the checklists were used to include or exclude studies or weight studies according to the appraisal of their quality.  The paper states that, all studies reporting outcomes on patient acceptability were included. “Data from included studies were extracted by one reviewer and checked by a second using a standardized data extraction form.” | *Type*  The synthesis takes the form of a charting of the results of indices of acceptability (as indicated below).  *Method*  Acceptability was measured in terms of:  (i) take-up rate (number of pts who started treatment/number of patients offered cCBT);  (ii) drop-out rate (number of pts who began cCBT but left before completing the cCBT course);  (iii) reasons for drop-out;  (iv) questionnaire or self-reported assessment of acceptability and satisfaction |
| Knowles 2014 | *Objective*  What are the common themes across qualitative studies about users’ experiences of computerised therapy; and how can these themes be used to improve the design of the next generation of programmes  *Type of review*  A systematic review and meta-synthesis of qualitative studies | *Search terms*  Derived from existing reviews including the Waller and Gilbody review. Population terms such as depression were combined with intervention terms such as technology assisted psychological therapy and qualitative research. One unpublished study (by the authors of this review) was included.  *Number and type of databases searched*  Five databases (eg Medline, PsycINFO, CINAHL, EMBASE and the Cochrane Library) and the Association of Computer Machinery (ACM) Digital Library. The conference abstracts of the Computer Human Interaction ACM special interest group were hand searched.  Search years range of each database searching - 2000 to September 2012  Publication year range of included studies – 2005 – 2012  *Number of studies included*  8  *Study design*  Qualitative studies (only)  *Setting/*  *context or geographic location*  Not reported in the results section of the paper. However, the eligibility criteria stated clearly that only minimal professional support was permitted and that, “…intervention[s]…exclusively delivered by a health professional who used technology to communicate with patients were excluded.”  It appears that the computer programmes could be accessed from a patient’s/service user’s home or a health setting (eg primary care) | *Type of mental health problems*  Mainly depression (Table 1 provides a list of mental health professionals that were excluded from the review – essentially comprising all MHPs except depression and anxiety).  7/8 studies refer to depression as the primary mental health problem. Participants in 1/8 studies had panic disorder. 1/7 studies had multiple sclerosis with depression and users in another 1/8 studies had depression and/or anxiety.  The extent to which depression and other mental health problems were diagnosed formally is unclear. However, the relevant heading in the table about study characteristics is labelled, Patient group, and the column describes computerised therapy users in several studies as, Primary care patients.  The number of participants across the 8 qualitative studies summed to 200 (127 women) and ranged from 8 (7 women) to 83 (47 women).  Age  6/8 studies range: 19-69 yrs, range of stated average age: 39-51 yrs;  2/8 studies comprised teenagers and young adults: range 14-21 yrs, average age 16-17yrs.  Gender  6/8 studies range: women 7-25, men 1-11;  2/8 studies (< 21yrs) range: 9-47, 4-36.  Ethnicity was reported in only 4/8 studies (1/4 studies comprised teenagers and young adults).  3/8 studies mostly White (9 Anglo-Australia & 1 Chinese-Australia; 8 White, 5 Black, 2 Hispanic; 35 White British, 1 Other White);  1/4 studies (< 21yrs): 38% Caucasian, 23% African-American, 6% Asian, 5% Hispanic, 4% Other. | Any “…psychological therapy… [not only CBT]…designed to bring about modifications of feelings, cognitions, attitudes and behaviour...delivered predominantly or solely by technology….”  7/8 studies referred to desktop computer-accessed programmes (eg Beating the Blues and MoodGym) and 1/8 studies investigated service users with depression who attended a psychiatric clinic and received ‘automated telephone-linked communication for depression’ that employed adherence and self-care training rather than CBT per se (and additional support in the form of clinic appointments). 6/8 studies used CBT - the only other study that did not use CBT per se was a study comprising people < 21 years – the study employed a 6-week CBT-Humanistic-Interpersonal Training Programme.  Staff support needed: 4/8 studies specified that additional support was provided: psychiatric clinic appointments, email support, group therapy, one f-to-f contact and up to 2 phone calls.  Supplementary support to cCBT intervention: Technical support was available in 1/8 studies (primary care pts with depression). | *Instrument*  A specific critical appraisal instrument was not used (see next section).    *Rating of quality analysis*  The reviewers appeared to conduct an appraisal (not on methodological criteria but) based on whether or not a paper contained sufficiently ‘thick’ data (not only descriptive data) about users’ experiences of technology-based therapy. Each study was required to report data that was collected via at least semi-structured interviews and to contain the results of at least a thematic analysis in order to be included in the review. | *Type*  Meta-ethnography and ‘line of argument’ synthesis (as indicated below).  *Method*  Participant quotes (1st order constructs) and the authors’ interpretations of participants’ quotes expressed as themes (2nd order constructs) were extracted from each paper onto an Excel sheet (by 2 reviewers and checked by a 3rd reviewer). Reviewers independently reviewed how the themes (2nd order constructs) juxtaposed and compared across papers and compiled 3rd order constructs that summarised and encompassed the various themes. Reviewers discussed and refined these constructs until a consensual understanding was reached - 3rd order constructs represent a synthesis of constructs that emerged from the analysis of 1st and 2nd order constructs.  Synthesis was achieved via a “…line of argument’…[approach which]…used the similarities and differences across the studies to develop an integrating scheme, or a ‘whole’ that made sense of the parts…[This analysis]…showed consistent themes but also apparent contradictions regarding users’ experience of computerised therapy and, therefore, the line of argument approach was utilised to make sense of apparent contradictions in the data and to integrate the emergent concepts to propose a model of user experience.” |
| Melville 2010 | *Objective*  To review the evidence to determine the extent of dropout from internet-based treatment for psychological disorders and factors associated with this drop-out. To explore the variables that put individuals at risk of drop-out in order to identify target groups and strategies to address drop-outs.  *Type of review*  A critical systematic review | *Search terms*  The following concepts: Internet, intervention and drop-out  *Number and type of databases searched*  PubMed and PsycINFO  *Other sources of study identification*  Citation lists of included studies  Search years range of each database searching – 1990-2009  Publication year range of included studies – 2000-2007  *Number of studies included*  19 studies  *Study design*  Unspecified  *Setting/*  *context or geographic location*  Unspecified | *Type of mental health problems*  Distress or dysfunction associated with DSM-IV or ICD-10 psychiatric disorders.  Age – Adults (unspecified age)  Gender – unspecified  Ethnicity –not reported;  SES – unspecified  Computer literacy – not reported;  Exclusions – Children, adolescents, people at-risk of psychological disorders, people with medical problems | “Internet-based therapies involving minimal therapist contact”  “…structured psychological treatments which the participant undertook more or less independently on the Internet (i.e. wither limited or no therapist support)…aimed at relieving distress or dysfunction associated with psychiatric disorders of adults”  “Interventions involving face-to-face therapist contact beyond a clinical interview, ongoing exchanges beyond emails, discussion forums, or scheduled telephone calls were excluded”  Staff support needed Unspecified  Supplementary support to iCBT intervention: Unspecified | *Instrument*  Not undertaken  *Rating of quality analysis*  Not applicable | *Type*  Narrative synthesis  *Method*  Unspecified |
| Rost 2017 | O*bjective*  To review literature on user acceptance of cCBT for depression over the last 10 years (2007-2016). Which measures were used to examine the user acceptance of cCBT for depression? What degree do users accept cCBT for depression?  *Type of review*  A systematic review | *Search terms*  Internet, Cognitive Behavioural Therapy, Acceptability and Depression  *Number and type of databases searched*  Web of Science, PubMed, CENTRAL and PsycINFO  *Other sources of study identification*  Citation lists of included studies  *Search years range of each database searching* 2007-2016  *Publication year range of included studies*  2009-2015  *Number of studies included*  29 studies  *Study design*  RCTs (16), Non-comparative studies (8), Qualitative (3), Comparative non-randomised study (2). One study compared two cCBT interventions and one study compared a guided and unguided cCBT.  *Setting/*  *context or geographic location*  Unspecified | *Type of mental health problems*  A diagnosis of depression of all degrees of severity  Age – Range 13 to 88 years old  Gender – Range 48-100% female  Ethnicity –not reported;  SES – unspecified  Computer literacy – not reported;  Exclusions – unspecified | “All cCBT interventions and their subtypes (e.g. mindfulness-based cognitive therapy and behavioural activation) delivered alone or as part of a package of care via the Internet”  Branded programs1 – MoodGYM (5), Beating the blues (4), Rainbow SPARX (2), ePST (2), The Sadness Program (2), Mom-Net (1), The Journey (1), Deprexis (1), Mobilyze (1), The Brighten Your Mood Program (1), Mom Mood Booster (1), Colour Your Life (1), Managing Your Mood (1), Mindful Mood Balance (10), Happy@Work (1), SHADE (1), Depression Free (1)  Staff support needed Nontherapeutic only (7), Therapist only (3), both (4), neither (3), unclear (9)  Supplementary support to iCBT intervention: Not specified | *Instrument*  Not undertaken  *Rating of quality analysis*  Not applicable | *Type*  Narrative synthesis  *Method*  Studies were assigned a score based on ‘levels of acceptance’: low (- -) 0-25%, moderate (-) 26-50%, high (+) 51-75% and very high (++) 76-100%. If evidence was inconclusive (i.e. presented both positive and negative aspects of acceptance) they were characterised (~) |
| Twomey 2017 | *Objective*  To investigate the effectiveness of the cCBT programme, MoodGYM for reducing symptoms of depression, anxiety and generalised psychological distress in adults. To conduct subgroup analysis addressing issues relating to cCBT.  *Type of review*  A systematic review | *Search term*  MoodGYM  *Number and type of databases searched* EMBASE, Medline, PsycINFO, CINAHL and Social Science Citation Index  *Other sources of study identification*  Citation lists of included studies  *Search years range of each database searching*  inception to 2016  *Publication year range of included studies*  2008-2014  *Number of studies included*  12 studies  *Study design*  RCTs (12)  *Setting/*  *context or geographic location*  Unspecified | *Type of mental health problems*  Elevated mental ill health symptoms seeking mental health intervention  Age – Adults mean age range 19 to 42 years old  Gender – Range 52-82% female  Ethnicity –not reported;  Socio-economic status – unspecified  Computer literacy – not reported;  Exclusions – unspecified | MoodGYM Including the following ‘modules’: introduction (fictional characters, core concepts and explanation of MoodGYM), feelings (negative though patterns, biased perceptions, negative views of self and future and link between thoughts and feelings), thoughts (identify and challenge biased thoughts, identify areas of vulnerability, self-esteem, pleasant activity scheduling diary), unwarping (talking out of dysfunctional thoughts, thought experiments, increase social and physical activity), de-stressing (learning about stress and identifying stressors, relaxation techniques), relationships (link between thoughts, emotions and relationships, problem-solving skills) and review.  Staff support needed: MoodGYm delivered with or without clinician support  Supplementary support to iCBT intervention: telephone (4), guided sessions (3), emails (2), guided sessions and emails (1), GP care (1), none (1) | *Instrument*  Cochrane Collaboration Risk of Bias tool for RCTs random sequence generation, allocation concealment and completeness of outcome data  *Rating of quality analysis*  Overall, quality was mixed. At least two of three criteria (8) and one criterion met (4)  Random sequence generation – + (8), - (4)  Allocation concealment – + (4), - (8)  Completeness of outcome data – + (11), - (1) | *Type*  Meta-analysis  *Method*  Pooled mean effect sizes hedge’s g (low 0.2, moderate 0.4 and large 0.8) and 95% CI using random effects models using Comprehensive Meta-analysis. Subgroup analysis include type of control group (no treatment versus active control), level of physician guidance (in-session versus remote versus none), setting (clinical versus non-clinical), country of trial (Australia versus Europe), conflict of interest (MoodGYM developer as co-author versus no conflict of interestI) and adherence to MoodGYM (average 50% sessions completed versus less than 50% of sessions completed on average). |
| Vallury 2015 | *Objective*  To synthesise global evidence on the effectiveness and acceptability of computerised CBT (cCBT) in the prevention or treatment of anxiety and depression for individuals who live in rural areas.  *Type of review*  A systematic review | *Search terms*  Variations of anxiety, depression, cognitive behaviour therapy, computerised cognitive behaviour therapy, online, application, health, computerised and eHealth.  *Number and type of databases searched* EMBASE, Medline, PsycINFO, CINAHL, Web of Science, Scopus and CENTRAL  *Other sources of study identification*  Citation lists of included studies and trial protocols  Search years range of each database searching – inception to 2014  *Publication year range of included studies*  2007-2013  *Number of studies included*  11 studies  *Study design*  RCTs (4), quasi-experimental (4); systematic review (1), Qualitative (1) and mixed methods (survey and qualitative) (1)  *Setting/*  *context or geographic location*  Unspecified | *Type of mental health problems*  Generalised or social anxiety disorders, multiple forms of anxiety and/or depression. Studies which included a number of anxiety disorders were included  Age – 16 and older (5), adolescents (3), young adults (18-25 years old) (1), unspecified (3)  Gender – Range 25-75% female  Ethnicity –not reported;  SES – unspecified  Computer literacy – not reported;  Exclusions – Studies focussing on individual phobias, post-traumatic stress disorder or postnatal depression | “CBT delivered via the Internet, through the use of a computer or other mobile electronic device”  The type, range and content of iCBT interventions or programmes were unspecified. Specific e-programs included ‘MoodGYM’ (4 studies), ‘FearFighter’ (2 studies) and ‘CRUfAD’ (2 studies), ‘SPARX’ (1 studies).  Staff support needed: unspecified  Supplementary support to iCBT intervention: unspecified | *Instrument*  Cochrane Collaboration Risk of Bias tool for RCTS and Grades of Recommendation, Assessment, Development and Evaluation (GRADE) criteria. Systematic review not appraised.  *Rating of quality analysis*  Collectively, studies had a moderate risk of bias.  RCTs- Low RISK OF BIAS (2), Moderate RISK OF BIAS (2)  Quasi-experimental (including mixed methods)- Unclear RISK OF BIAS (1), moderate (1) and low (3)  Qualitative- Moderate RISK OF BIAS (1)  Review sought only published studies so potential risk of publication bias. | *Type*  Narrative synthesis  *Method*  Method of narrative synthesis grouped studies according to the following outcomes: efficacy, uptake and referral, adherence/attrition and other measures of acceptability |
| Waller 2009 | *Objective*  To examine barriers to the uptake of cCBT in terms of the acceptability, accessibility and adverse consequences associated with cCBT  *Type*  A systematic ‘integrated’ review (qualitative data and quantitative data from individual eligible studies were integrated) | *Search terms*  Anxiety or depression, psychotherapy (specifically CBT) and computerised delivery. Names of cCBT packages were also searched as text words.  *Number and type of databases searched* Biology Abstracts, CINAHL; CENTRAL; CDSR; EMBASE, HMIC Medline, Medline Plus, DARE; NHS EED; PsycINFO, Science Citation Index, Social Sciences Citation Index.  *Search years range of each database searching* Inception to July 2005  *Publication year range of included studies*  1984 – 2005  *Number of studies included*  36  *Study design*  RCTs (18), non-RCTs (5), cohort study (4), survey (5), qualitative (4).  *Setting/*  *context or geographic location*  Unspecified – there are some references to university centres and (UK) GP Practices. Countries – unspecified. However, the search terms included primary and secondary care as well as the internet. | *Type of mental health problems*  Anxiety, depression or both -17/3 studies; Phobias, panic disorder or both – 14/32 studies; stress 1/32 studies.  Quantitative data: Age – not reported; gender – not reported; ethnicity –not reported; SES – 66% employed, 16% university students (4 studies), a further 2 studies comprised university students only; computer literacy – 35%-62%; exclusions – high risk clients excluded eg 255 people from 7 trials excluded due to risk of suicide  Qualitative data: Age – not reported; gender – not reported; ethnicity –not reported; SES – ‘socially deprived’ (3 studies), mixed urban/rural (1 study), ‘a range of social classes’ (1 study); computer literacy – training or familiarisation with cCBT (8 studies); exclusions – people who could not read or write (Discussion section stated that typical CBT language has a reading age of 17 years old and, therefore, poses a potential barrier). | “Computerised cCBT, defined as: a fully or partly computerised environment where the computer directed therapy (for example, taking a lead on formulation, risk management, and suggesting interventions). Studies where the therapist delivered the CBT and was only assisted by the computer were excluded – for example, online Bibliotherapy, e-mail therapy/counselling, virtual reality for exposure.” (This is the description in PICO reported in supplementary data on journal website).  The type, range and content of cCBT interventions or programmes in the included papers were not specified (service users’ (largely) positive views are reported below) nb. I think that this lack of specification and ‘lumping’ is a major weakness in the various reviews.  Staff support needed: Quantitative data – 0 to 150 mins/client (one study) to 3.7 times less than therapist-led CBT; type of staff – not reported. Qualitative data – minimal (unspecified) support provided; type of staff – ‘supervised junior staff or qualified therapists or psychiatrists’  Supplementary support to cCBT intervention : Quantitative data – no additional data. Qualitative data – ‘...online discussion rooms, email reminders, bibliotherapy and lengthy handbooks.’ (number of studies unspecified); cCBT was situated within ‘stepped-care’ mental health provision in only 2 studies. | *Instrument*  Quantitative - the Cochrane Effective Practice and Organisation of Care Group (Bero, Grilli et al. 1998) criteria; Qualitative - Mays and Pope (Mays 1996) criteria; Surveys Crombie criteria (Crombie 1996)  *Rating of quality analysis*  Not reported. However, the Discussion section of the review states that there was variation in methodological quality and “...the sources for qualitative data [were] generally poor” with more weight being assigned (in an unspecified way) to higher quality studies. | *Type*  Integrated (combined quantitative-qualitative) synthesis and REM synthesis.  *Method*  According to the following outcomes: efficacy, uptake and referral, adherence/attrition and other measures of acceptability |
| Zhou 2016 | *Objective*  To evaluate the evidence regarding the effectiveness of iCBT versus control groups on improving symptoms of subthreshold depression  *Type of review*  A systematic review | *Search terms*  Subclinical depression, iCBT and randomised controlled trial  *Number and type of databases searched* CENTRAL, Medline, PubMed, Web of Science, ScienceDirect and PsycArticles  *Other sources of study identification*  Citation lists of included studies  *Search years range of each database searching* 2005 to 2016  *Publication year range of included studies*  2007-2016  *Number of studies included*  8 studies (10 articles)  *Study design*  RCTs (9); Control groups- Waitlist (5 studies, 6 articles), Attention control (2 studies, 3 articles), Waitlist and Attention control (1 study)  *Setting/*  *context or geographic location*  At home or other places with internet access. | *Type of mental health problems*  Subclinical depressive symptoms-- Participants with elevated depression on a standardised depression inventory  Age – Employees over 18 years old (4 studies), Elderly patients (3 studies), Adults over 18 years (2 studies), not reported (1 study);  Gender – not reported;  Ethnicity –not reported; SES – 44% employed (4 studies), not reported in remaining 6 studies;  Computer literacy – not reported;  Exclusions – high risk patients e.g. diagnoses of major depressive disorder, other primary mental disorders, alcohol and drug dependency and reports of psychotic symptoms or suicidal ideation. | “ICBT programs were defined as interventions based on theories of cognitive behavioural therapy, not conducted in a clinic, and delivered to participants via the internet” (This is the description in ‘Types of intervention’ reported in methods section).  The type, range and content of iCBT interventions or programmes in the included papers included psychoeducation, cognitive restructuring, behavioural activation and other related skills. iCBT lasted between three and 10 weeks with one or two sessions a week. Specific e-programs included ‘Coping with depression’ (1 study), ‘MoodGYM’ (1 study), ‘The UniWellbeing Course’ (1 study) and ‘GET.ON Mood Enhancer’ (1 study).  Staff support needed: Therapist-led including emails, phone-calls, feedback and tailored treatment (6 studies) and self-help (4 studies).  Supplementary support to iCBT intervention : Online discussions (number of studies unspecified) | *Instrument*  Cochrane Collaboration Risk of Bias tool (see Fig. 2) - Selection bias (sequence generation, allocation concealment), Performance bias (blinding of participants, personnel), Detection bias (blinding of outcome assessors), Attrition bias (Incomplete outcome data), Reporting bias (selective outcome reporting) and other threats of validity (unspecified).  *Rating of quality analysis*  Independent assessment undertaken by two authors with disagreement resolved through group discussion. Selection bias (Low Risk of Bias), Performance bias (Unclear), Attrition bias (Low Risk of Bias in most studies), Reporting bias (Low Risk of Bias in most studies) and Other threats of validity (Unclear in most studies, but unspecified what sources of bias). | *Type*  Quantitative data from RCTs  *Method*  Meta-analysis was conducted at the study level using RevMan 5.1. Choice of fixed or random effects model was dependent on homogeneity analysis results. Weighted mean difference or standardised mean difference was used depending on similarity of outcome measures. Method of narrative |

^a^Numbers in parentheses refer to n of studies, some studies included more than one intervention arm
