# Supplementary material for: Acceptability of Computerized Cognitive Behavioral Therapy for Adults: Umbrella Review
Source: JMIR Ment Health. 2021 Jul 6;8(7):e23091. doi: 10.2196/23091 (PMC8292944; doi:10.2196/23091)
Supplement: Multimedia Appendix 3 [file mental_v8i7e23091_app3.docx]

**Supplementary Table 4: Summary of satisfaction or acceptability findings**

| Satisfaction or acceptability of cCBT components or mode of delivery | Author/year | Intervention (population) | N studies/participants | Results/findings (number of studies) |
| --- | --- | --- | --- | --- |
| Overall satisfaction/acceptability levels | Kalthenthaler 2008 [31]  Waller 2009^a^ [24]  Vallury 2015 [23]  Rost 2017 [25] | Computerised CBT  (Mild or moderate depression)  Computerised CBT  (Common mental health disorders)  Computerised CBT (Depression and anxiety)  Computerised CBT  (Depression) | 6 studies  4 studies  1 study  8 studies | Overall satisfaction/acceptability with cCBT was high (6), participants were happy to use cCBT (92% versus 3% not happy) (1), would recommend to others (1), participants expressed high levels of satisfaction with the clinic as a whole (1)  98% users satisfied with cCBT (1), 60% good to very good satisfaction with cCBT (1), Would recommend cCBT to a friend (1) ^b^, Users would pay £10 per session (1)  Rural participants more likely to report that cCBT helped their depression and substance misuse compared to urban participants (92 versus 75%), 97% of rural participants were satisfied with support for cCBT, two-thirds also thought increased autonomy and confidentiality were benefits of cCBT, privacy was reported to be an important feature of cCBT (1)  Very high (6(1)^b^), Moderate (1), Mixed reports (1) |
| Remote accessibility | Waller 2009 ^a^ [24]  Melville 2010 [27]  Beatty 2016 [26]  Rost 2017 [25] | Computerised CBT  (Common mental health disorders)  Internet-based treatment  (Psychological dysfunction or distress related to psychiatric conditions)  Self-directed psychological intervention  (Psychological outcomes for mental and physical disorders)  Computerised CBT (Depression) | 2 studies  1 study  1 study  3 studies  7 studies  2 studies | - Can do in own time and at home (2(1) ^b^) - Users are less compliant with more time on study, despite prompts (1)   Lack of peace and quiet for computerised environment  Positive perceptions associated with increased adherence   - Can complete in own time   Negative perceptions associated with non-adherence   - Lack of time - Too busy - Privacy   Reasons for drop-out   - Lack of motivation - Lack of time - Too busy - Too time-consuming   Both positive and negative views were expressed with regards to *anonymity* (+Reduced pressure compared to face-to-face CBT, -cCBT increased isolation and loneliness) and *flexibility* (+High degree of control, use cCBT as and when wanted (2), -Easy to avoid and difficult to sustain) of cCBT (1) |
| Computerised delivery | Kalthenthaler 2008 [31]  Waller 2009 ^a^ [24]  Melville 2010 [27]  Beatty 2016 [26]  Rost 2017 [25] | Computerised CBT  (Mild or moderate depression)  Computerised CBT  (Common mental health disorders)  Internet-based treatment  (Psychological dysfunction or distress related to psychiatric conditions)  Self-directed psychological intervention  (Psychological outcomes for mental and physical disorders)  Computerised CBT  (Depression) | 3 studies  5 studies  4 studies  2 studies  1 study | Easy to use (84% versus 3%) (1), comfortable using cCBT (1), technical aspects (1)  9% of participants would not use a PC, -Older users were more likely to drop out and took slightly longer to use cCBT (due to IT skills) (1*), Drop-out reportedly due to technical difficulties (1) ^b^, cCBT seen as discriminatory against low IT skills (1), +PC literacy not related to drop-out (1), +Training appropriate (1), treatment satisfactory (1), +Technical aspects rated as good to moderate (1)  Lack of reliable computer access, lack of computing experience, PC and Internet issues, a dislike of computers  Poor computer literacy associated with non-adherence (1), Computerised delivery perceived as too stressful or error-ridden (2)  Inadequate computer and Internet skills was reported as a barrier to cCBT (1) |
| Level of guidance | Kalthenthaler 2008 [31]  Waller 2009 ^a^ [24]  Melville 2010 [27]  Vallury 2015 [23]  Beatty 2016 [26]  Rost 2017 [25] | Computerised CBT  (Mild or moderate depression)  Computerised CBT  (Common mental health disorders)  Internet-based treatment  (Psychological dysfunction or distress related to psychiatric conditions)  Computerised CBT (Depression and Anxiety)  Self-directed psychological intervention  (Psychological outcomes for mental and physical disorders)  Computerised CBT  (Depression) | 5 studies  6 studies  1 study  1 study  9 studies ^b^  4 studies ^b^ | iCBT was considered acceptable though not all sessions were completed and iCBT was less popular than counselling (1), preference for guided versus self-guided cCBT (2), live support (1)  Therapist viewed as more helpful than cCBT (1); users were fairly satisfied with cCBT, but more satisfied with face-to-face therapy (1), marginal preference for therapist, no difference in satisfaction between cCBT and therapist (1), cCBT better than previous experience with treatment (1) ^b^, Role of therapist: Complex cases better managed by therapist (1), appreciates remote delivery and not meeting therapist (1), psychiatrist were more tolerant (1), live help perceived to be more helpful and credible (1), users perceived staff to have poor knowledge of cCBT programme (1) ^b^  Preference for face-to-face treatment was stated as a reason for dropping out of cCBT  Rural versus urban participants less likely to want face to face contact (18 versus 48%) (1 study), Two-thirds of rural participants missed therapist contact  Lack of guidance during cCBT perceived as lacking human contact and absence of feedback was associated with low adherence (9)  No difference in satisfaction between participants in guided and unguided cCBT (1), participants in guided cCBT were more satisfied than participants in unguided cCBT (1), participants in unguided cCBT reported it was easy to use and to have longer lasting effects compared to users in guided cCBT (1), Having added support improved adherence (1) , absence of support led to a failure to gain understanding and acted as a barrier (1) |
| cCBT content | Kalthenthaler 2008 [31]  Waller 2009 ^a^ [24]  Beatty 2016 [26]  Rost 2017 [25] | Computerised CBT  (Mild or moderate depression)  Computerised CBT  (Common mental health disorders)  Self-directed psychological intervention  (Psychological outcomes for mental and physical disorders)  Computerised CBT  (Depression) | 4 studies  8 studies  3 studies  1 study | Booklets were viewed as helpful (2), multimedia delivery was acceptable for 91% of users (1), content structure (1)  Newer programmes have better adherence (1), Like design and delivery (1), Programme viewed as a ‘cold’ interface (1), clients unaware of true nature of therapy upon starting (1), cCBT programme: +/-preferred cCBT programme orientated to faith, +cCBT very logical (1) ^b^, +more users preferred cCBT (44%) to therapists (12%), 44% neutral, +easy to understand (100% of users), 80% cCBT better than book (1), content viewed as good to moderate (1*), support viewed as good, very good (1*), easy to use (most users) (1), all users felt understood by cCBT (1), -Too demanding (1) ^b^, pace too fast (1) ^b^, emails impersonal (1) ^b^, programme found to be boring after first few sessions (1), small problems experienced when undertaking cCBT in public place (1), case studies and content viewed as patronising or unrealistic at times (1)  When perceived as helpful and beneficial was associated with increased adherence (1), when cCBT was perceived as impersonal and irrelevant was associated with low adherence (3)  Lack of identification with programme was reported as a barrier to cCBT (1) |
| Circumstances | Waller 2009 ^a^ [24] | Computerised CBT  (Common mental health disorders) | 1 study | Advantage to cCBT being delivered in primary care, GP referrals were best, users who self-referred were most motivated, users with mental health referrals were less motivated (1) |

^a^Overlapping studies

^b^Study grouped qualitative data under three headings referring to *Therapy, Circumstances* and *Technology*
