# Supplementary material for: Acceptability of Computerized Cognitive Behavioral Therapy for Adults: Umbrella Review
Source: JMIR Ment Health. 2021 Jul 6;8(7):e23091. doi: 10.2196/23091 (PMC8292944; doi:10.2196/23091)
Supplement: Multimedia Appendix 4 [file mental_v8i7e23091_app4.docx]

**Supplementary Table 4: Qualitative findings**

| User, carer or health-care professionals on their cCBT experience | Author/year | Intervention (population) | N studies/participants | Results/findings (number of studies) |
| --- | --- | --- | --- | --- |
| The desire for computerised therapies to be responsive to ‘self’ | Knowles 2014 [30] | Computerised therapy  (Depression and anxiety) | 8 studies | ‘Who I am’ as a patient, including clinical needs (such as physical comorbidity) and personal preferences (with users requesting reactive, personalised content); ‘How I Feel’, recognising the demands of depression on the user (such as emotional and motivational difficulties, and problems with concentration) |
| The dialectical nature of user experience | Knowles 2014 [30] | Computerised therapy  (Depression and anxiety) | 8 studies | ‘Level of support’: *Empowerment* and *Burden*; ‘Contact with others’: *Anonymity* and *Burden* |
| Experiencing unrelated personal reasons | Beatty 2016 [26] | Self-directed psychological intervention  (Psychological outcomes for mental and physical disorders) | 1 study | Associated with non-adherence |
| Symptomatology of mental illness | Beatty 2016 [26] | Self-directed psychological intervention  (Psychological outcomes for mental and physical disorders) | 2 studies | Symptomatology of depression (i.e. motivation and concentration) acts as a barrier to adherence to cCBT (1), improvement in condition was associated with non-adherence as individuals felt they were sufficiently helped |
| Participation in cCBT | Kalthenthaler 2008 [31]  Vallury 2015  Beatty 2016 [26] | Computerised CBT  (Mild or moderate depression)  Computerised CBT (Depression and anxiety)  Self-directed psychological intervention  (Psychological outcomes for mental and physical disorders) | 3 studies  1 study  4 studies | Perceived benefits of participation include: improved QoL (1), good for helping with anxiety and/or depression (1). Perceived negatives include: no long-lasting effects (1)  92% of urban users compared to 75% of rural users believed cCBT helped them with depression and substance misuse  Associated with adjustment (1), 10 participants discontinued cCBT as it was perceived to be too intensive (1) or too general or too limited (1), or having a negative experience with specific components (2) |
| Therapist/Healthcare professional views | Waller 2009 [24]^a^ | Computerised CBT  (Common mental health disorders) | 1 study | *Circumstances*   - Need for institutional support to fund costs for cCBT - Need for a dedicated computer room   *Therapy*   - Need for a British version - 13% of therapist reported cCBT more likely to harm than therapist, 16% of therapists reported cCBT no more likely to harm than therapist - No therapist believed that cCBT was more effective than therapy, 36% of therapists believed that cCBT was no more effective than therapy - Therapists believed that cCBT was less effective for compliance, satisfaction and client expectation of success - cCBT is more beneficial than written self-help: yes 25% of therapists, no 5% of therapists - cCBT as an alternative to therapist: yes 27%, no 9% - 81% of therapists believed cCBT would be a supplementary to therapy   *Technology*   - Therapist would like more training in cCBT - Expressed concerns about data protection issues |

^a^Overlapping studies
